# A Microfluidic Approach for Profiling Total Nitrogen Content in Age-Specific Nutritional Formulas Using Microchip Gel Electrophoresis

**DOI:** 10.3390/ijms26178233

**Published:** 2025-08-25

**Authors:** Fruzsina Balogh-Hartmann, Csilla Páger, Anna Dávidovics, Sára Nagy, Tamás Marosvölgyi, Lilla Makszin

**Affiliations:** 1 Szentágothai Research Center, Institute of Bioanalysis, Medical School, University of Pécs, 7624 Pécs, Hungary; fruzsina.hartmann@aok.pte.hu (F.B.-H.); csilla.pager@aok.pte.hu (C.P.); marosvolgyi.tamas@pte.hu (T.M.); 2 Department of Languages for Biomedical Purposes and Communication, Medical School, University of Pécs, 7624 Pécs, Hungary; anna.davidovics@aok.pte.hu; 3 Doctoral School of Health Sciences, Faculty of Health Sciences, University of Pécs, 7621 Pécs, Hungary; nagy.sara@edu.pte.hu

**Keywords:** nutritional formulas, kidney disease, microfluidic profiling, total nitrogen content, microchip gel electrophoresis

## Abstract

Accurate assessment of protein content in Foods for Special Medical Purposes (FSMPs) is critical for patients with chronic kidney disease, who require tightly regulated protein intake. This study aimed to develop and apply a rapid, low-volume, and reproducible microchip-based gel electrophoresis method for analyzing total nitrogen (TN) content and electrophoretic profiles in FSMPs. Products of different consistencies (powder, liquid, yoghurt-like) were tested to evaluate the influence of common additives (e.g., milk proteins, stabilizers, sweeteners) on TN levels and protein patterns. The results revealed considerable variation in fractions among brands, largely attributable to additive composition. Notably, TN levels often exceeded the declared protein content, potentially leading to unintended nitrogen overconsumption in clinical settings. Statistical analysis identified significant TN differences between infant and adult FSMPs in liquid formulations, while powdered forms showed no such distinction. These findings highlight the clinical importance of precise analytical monitoring, as discrepancies between measured TN and labeled protein content could compromise dietary management in vulnerable populations. The proposed method provides a reliable tool for FSMP quality control and supports safer nutritional planning in therapeutic diets.

## 1. Introduction

This study focused on the determination of the electrophoretic profile and total nitrogen (TN) content of foods intended for specific groups, including Foods for Special Medical Purposes (FSMPs). Previously categorized as nutritional formulas, these products are now officially classified as “specialty foods”, according to a 2017 communication from the European Commission [1]. Therefore, from a food quality and safety perspective, proper testing of FSMP ingredients is a critical concern. Unlike medicinal products, FSMPs are not subjected to rigorous clinical testing prior to market release. Instead, they are placed on the market through a simplified notification procedure. Manufacturers are required to label ingredients on the packaging of their products in accordance with the applicable EU regulations and specified limits [2]. FSMPs are widely accessible and are not subject to prescription. As such, they are distributed not only through pharmacies but also via drugstores and retail shops, regardless of the specific target group they are intended for.

One focus of this study was on *FSMPs specially formulated for babies, newborns, and premature infants*. Breastfeeding is widely recognized for providing an optimal balance of essential nutrients, including proteins, fats, carbohydrates, vitamins, and minerals, as well as immune-related compounds that support infant health and proper development [3,4]. When breastfeeding is not possible, medical consultation is recommended to select the most appropriate FSMPs. Skimmed milk-based FSMPs, closely approximating breast milk’s composition, are generally advised unless there is a milk protein allergy. In such cases, hypoallergenic (HA) or amino acid-based FSMPs are recommended depending on allergy severity or feeding disorders [5]. With the global increase in milk protein allergies, HA formulas and reduced-protein products are being used more frequently, not only for treatment but also as a preventative option [6,7,8,9]. Regarding protein content, one of the primary components of skimmed milk-based FSMPs is casein, especially α-, β-, and κ-casein in standard formulas, and their hydrolyzed forms in HA versions. Infant FSMPs are designed to mimic human breast milk, which contains approximately 60% rapidly digested whey proteins and 40% casein [10].

FSMPs *for adults* are used to address compromised nutrient intake due to illness, injury, or increased physical demands such as athletic performance [11,12]. Many nutritional supplements are specifically formulated for older adults, who often require support in maintaining muscle mass and bone strength as they age [13]. These FSMPs are commercially available in a variety of forms, including powders, liquids, and yoghurt. Exceptions include clinical formulations administered enterally or parenterally in hospital settings under medical supervision [14,15]. Milk-based FSMPs have relatively consistent protein profiles, while plant-based alternatives (soy, rice, pea) serve individuals with milk protein allergies but may pose other allergen risks [16,17,18,19,20,21,22]. Many formulations also incorporate plant proteins, free amino acids, and peptide-based hydrolysates to meet specific nutritional needs, including those related to metabolic disorders or nutrient deficiencies, all of which contribute to the TN content [16,17,18,19,20,21,22,23].

To improve taste and texture, manufacturers frequently add various components to FSMPs [24], which also help ensure adequate nutritional needs of the target group [4,24,25]. Both protein-based and non-protein nitrogen sources can be distinguished, with each contributing to the TN content of the product [16,24]. The TN content of an FSMP may vary considerably depending on the target population, the physical form of the product, and the technological processes involved in its manufacture. One infant liquid FSMP we analyzed contains 2′-fucosyllactose (2′-FL), the most abundant oligosaccharide in human breast milk, known for its prebiotic, anti-inflammatory, and immune-supporting effects [3,9,26,27,28]. Other important components influencing nitrogen content, stability, and bioavailability include stabilizers and emulsifiers like pectin and lecithin [29,30], essential vitamins, particularly B1, B9, and B12 [18,19,24], and maltodextrin, frequently listed as a foaming agent on FSMP labels, may indirectly contribute to nitrogen content [31]. Minor nitrogen sources can also include colorants, flavor enhancers, and both natural and artificial sweeteners [24].

Protein intake is a particular concern in patients with chronic kidney disease (CKD) [32]. Protein metabolism produces nitrogenous waste like ammonia, creatinine, and uric acid, which are cleared by the kidneys. In healthy individuals, the glomerular filtration rate (GFR) ranges from 100 to 120 mL/min, enabling effective waste removal. CKD impairs this filtration, causing nitrogenous waste to accumulate in the blood. CKD is classified into five stages based on GFR [33], and dietary protein restriction is crucial at all stages to prevent further kidney damage [32,33,34]. Excessive nitrogen accumulation can contribute to the development of uremia, a pathological condition characterized by the retention of waste products in the blood, which may manifest with symptoms such as fatigue, nausea, and, in severe cases, multi-organ dysfunction [32]. Therefore, careful monitoring of nitrogen intake is essential in CKD patients, as nitrogen originates from multiple dietary sources, primarily proteins and certain food additives, both of which can collectively increase the body’s TN load [32,33].

Protein and nitrogen content in nutritional products can be determined using either direct or indirect methods. Among the indirect techniques, the Kjeldahl and Dumas methods are the most widely used for estimating total nitrogen [35,36]. While these methods are well-established, they have notable limitations: they require extensive sample preparation and use hazardous reagents. Given these constraints, there is increasing interest in alternative analytical platforms that allow for both total nitrogen quantification and molecular-level profiling. Microchip gel electrophoresis, especially when combined with fluorescence labeling, offers a rapid, reproducible, and miniaturized solution for assessing nitrogen-containing compounds in complex matrices. Consequently, there is increasing demand for microfluidic-based analytical technologies in the field of food and nutritional analysis.

Based on this approach, the present study was designed with the following objectives: (i) to apply microchip gel electrophoresis using the High Sensitivity Protein 250 (HSP 250) method for the efficient analysis of FSMPs produced by different manufacturers; (ii) to determine the total nitrogen content of each product and compare these values with the declared label information; (iii) to compare the complete electrophoretic profiles of FSMPs in various forms, including powders, liquids, and yoghurts; and (iv) to evaluate the differences in electrophoretic profiles between infant and adult FSMP products with similar consistencies. These comparative analyses may reveal meaningful differences, providing important information for individuals with chronic kidney disease, for whom accurate monitoring of protein intake is essential.

## 2. Results and Discussion

### 2.1. Total Nitrogen Content Comparison

In this study, a total of fifty-five FSMPs with different physical consistencies, such as liquid, powder, and yoghurt, were analyzed. These products, designed for different target groups, contain both protein and non-protein nitrogen (NPN) components. Since FSMPs are classified as food products for special medical purposes rather than pharmaceuticals, their mandatory labeling must comply with Council Regulation (EU) No 1169/2011 [2], which requires the declaration of total protein content (g/100 mL). In this study, both the protein profile and TN content (encompassing both protein-derived and NPN components) of FSMPs with different consistencies were assessed. When calculating TN concentration, factors such as the time-corrected area under the peak (TCA), ladder concentration, and dilutions used during sample preparation were considered. TN content was determined according to the original protocol [37].

Table 1 presents a comparison between the total nitrogen (TN) concentrations and the nominal total protein (TP) values indicated on the product labels. The TN contents of FSMPs measured by microchip gel electrophoresis were 9.3 (4.1–23.4) g/100 mL liquid FSMPs for newborns, 21.2 (9.4–45.2) g/100 mL liquid FSMPs for adults, 10.9 (7.3–54.8) g/100 mL powder FSMPs for newborns, 75.6 (38.8–81.4) g/100 mL powder FSMPs for adults, and 3.9 (3.7–12.6) g/100 mL yoghurt FSMPs for adults. The HSP 250 method allows for reproducibility of the peak area range within a coefficient of variation (CV) tolerance of ±20% for the calculation of TN content [38]. Reproducibility was evaluated by performing three replicate measurements on each sample. The relative standard deviation (RSD) ranged between 1.8% and 4.5%, which is within acceptable limits for comparable analytical techniques. Statistical analysis revealed significant differences between the total protein content declared on the product labels and the measured TN content for the FSMP categories: liquid FSMPs for newborns (*p* = 0.011), liquid FSMPs for adults (*p* < 0.001), powder FSMPs for newborns (*p* = 0.001), and powder FSMPs for adults (*p* = 0.026). However, no significant difference was found in yoghurt FSMPs for adults (*p* = 0.506). The statistical results showed that the measured TN content (measured TN) of all FSMPs exceeded the nominal label values (nominal TP) (Table 1), which could be attributed to the presence of protein and NPN additives. A list of protein and non-protein nitrogen (NPN) additives that may affect TN values is provided in Supplemental Table S1.

The Kruskal–Wallis non-parametric test was employed to evaluate statistically significant differences in TN content among samples with varying expiration dates. Analyses were conducted separately for each brand within each FSMP category. No statistically significant differences in TN content were observed across expiration dates within any brand or product category. The mean *p*-values and their respective ranges were as follows: adult liquids, *p* = 0.333 (0.212–0.675); infant liquids, *p* = 0.878 (0.742–0.902); powdered adult formulas, *p* = 0.902 (0.878–0.945); powdered infant formulas, *p* = 0.546 (0.488–0.760); and yoghurt-based adult FSMPs, *p* = 0.778 (0.745–0.812). The results indicate that TN content remains stable across different expiration dates within each brand, demonstrating a high degree of compositional consistency over time.

### 2.2. Comparison of FSMP Brands Within the Same Category

**Figure 1:**
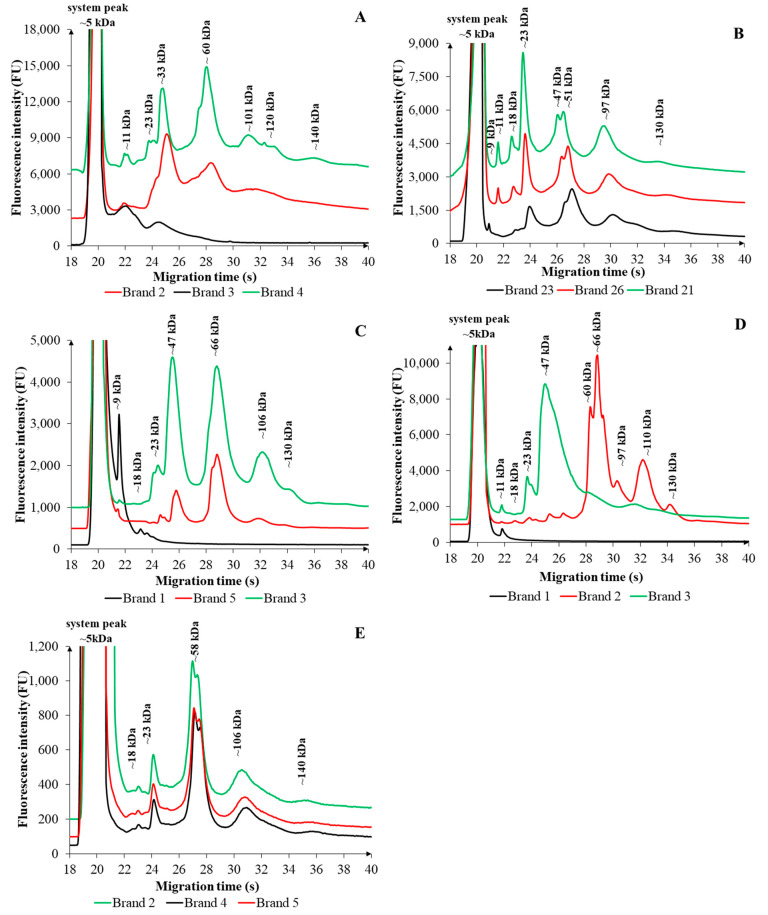
Electropherograms of five distinct categories (**A**–**E**) of FSMPs, each represented by three different commercial brands. (**A**) liquid FSMPs for newborns, (**B**) liquid FSMPs for adults, (**C**) powder FSMPs for newborns, (**D**) powder FSMPs for adults, (**E**) yoghurt FSMPs for adults. The observed migration patterns reflect the characteristic profiles of protein and non-protein nitrogen (NPN) components specific to each formulation. The experimental conditions are described in Section 3.

Figure 1 displays representative electropherograms of FSMPs with different consistencies, derived from the three different brands. Each profile highlights the protein and non-protein nitrogen (NPN) additive patterns typical of the respective product categories. The experimental conditions used for the microchip gel electrophoresis (MGE) analysis were optimized and are described in detail in Section 3.

For each FSMP type, three brands were selected based on the prevalence and diversity of additive content to capture the characteristic profiles of formulations intended for specific target groups.

In the case of infant-formulated liquid FSMPs (Figure 1A), different brands displayed distinct electrophoretic profiles. Brand 4 exhibited the most complex protein pattern, with fractions observed at molecular weights of approximately 11, 23, 33, 60, 101, 120, and 140 kDa, respectively. The protein profile of Brand 2 was similar in composition to that of Brand 4, although it featured fewer peaks, predominantly of lower intensity, appearing at approximately 11, 33, 60, and 101 kDa. In contrast, Brand 3 demonstrated a clearly different protein profile; among the four visible fractions, ~11 kDa displayed the highest fluorescence intensities.

Several core ingredients were common to all three tested brands, including lactose, whey protein or its hydrolysates, fish oil, and vegetable oils such as coconut, sunflower, and rapeseed oil. All brands also contained vitamin B12, which, along with other components, may contribute to the TN content of the formulas [16,24,39]. Brand-specific differences were also observed: soybean oil and lecithin were unique to Brand 4 and were not present in the other two formulations. Conversely, *Mortierella alpina* oil was present in Brands 2 and 3 but absent from Brand 4. Brands 2 and 3 also shared a similar additive profile, differing mainly in the inclusion of 2′-fucosyllactose (2′FL), which was exclusive to Brand 2. Brand 4 contained a relatively lower number of added compounds than the other two. Despite these compositional and electrophoretic profile differences, statistical analysis revealed no significant differences in TN content among the tested brands of infant liquid FSMPs at the sample number used (*p* = 0.072).

In the case of *adult-formulated liquid FSMPs* (Figure 1B), Brands 21, 23, and 26 were selected for illustration. The electropherograms revealed that Brands 21 and 26 shared highly similar electrophoretic profiles, both in terms of peak number and intensity. Distinct fluorescence peaks were observed at molecular weights of approximately 9, 11, 18, 23, 47, 51, 97, and 130 kDa. In contrast, Brand 23 displayed a slightly different profile, with clear differences in both the number of peaks and their relative intensities. All three FSMPs contained several common ingredients, including milk protein, maltodextrin, vegetable oils, flavorings, and emulsifiers (soy lecithin and E 471), which may account for the observed similarities in their electrophoretic patterns. Additionally, all products included B-group vitamins such as thiamine hydrochloride, folic acid (pteroylmonoglutamate), and cyanocobalamin. The minor differences may be attributed to the presence of fish oil exclusively in Brand 23 and the use of the sweetener acesulfame-K exclusively in Brand 26. Statistical evaluation of TN content revealed a significant difference between the brands (*p* < 0.001), likely reflecting variations in both the quantity and nature of additive compounds contributing to the total nitrogen content [16,23,24,29,31,39].

Among the *powdered FSMPs formulated for newborns* (Figure 1C), Brands 1, 3, and 5 were selected for comparative analysis. The electrophoretic patterns showed that Brands 3 and 5 exhibited similar profiles in terms of the number and position of the fractions, with the main difference being the relative intensity of the peaks. Notably, Brand 3 showed higher fluorescence intensities across all detected fractions. Both Brands 3 and 5 displayed six distinct fractions, with molecular weights of approximately 9, 23, 47, 66, 106, and 130 kDa. In contrast, Brand 1 showed a much simpler pattern, dominated by a single prominent peak at ~9 kDa. The compositions of the three FSMP products were broadly similar, with all products containing milk-derived proteins (e.g., whey protein or hydrolyzed whey protein), vegetable oils (such as coconut, rapeseed, and sunflower oil), fish oil, and a variety of B vitamins such as thiamine (B1) and folic acid. Moreover, each product included L-carnitine and multiple nucleotide additives (uridine-, cytidine-, adenosine-, inosine-, and guanosine-5′-monophosphate sodium salts), all contributing to the total nitrogen content. Notable differences were observed between the additives. Brand 1 was formulated exclusively with hydrolyzed whey protein, whereas Brands 3 and 5 also included additional milk-based ingredients, such as lactose, skim milk, and whey protein concentrate, which is consistent with the differences observed in their electrophoretic profiles. Brand 3 uniquely included cereal flakes (rice flour), whereas Brand 5 contained an additional thickening agent (carob bean gum). Soy lecithin was used as an emulsifier in all three brands. However, only Brand 3 contained vitamin B12, Brand 1 was uniquely supplemented with L-tyrosine, and both Brands 3 and Brand 5 contained L-tryptophan. Statistical analysis confirmed a significant difference in TN content among the brands (*p* = 0.021), attributable to variations in protein sources and nitrogen-contributing additives [16,22,24].

Among the *powdered FSMPs formulated for adults* (Figure 1D), Brands 1, 2, and 3 were selected for comparative analysis. The electropherograms revealed distinct profiles for each product, differing in both peak number and intensity. Brand 1 displayed a single dominant peak corresponding to a molecular weight of approximately 11 kDa. In contrast, Brand 2 presented the most complex electrophoretic profile, displaying ten fractions with molecular weights of approximately 11, 18, 23, 40, 49, 60, 66, 97, 110, and 130 kDa. Brand 3 showed four detectable fractions at ~11, ~23, ~47, and ~106 kDa. Comparative analysis of additive compositions across the three brands revealed both similarities and notable differences, corresponding with their electrophoretic profiles. Brand 1 was the simplest formulation, containing only maltodextrin. In contrast, Brands 2 and 3 included milk or milk-derived protein components, along with a spectrum of B-vitamins such as thiamine (B1), vitamin B12, and folic acid. Brand 3 was uniquely characterized by the inclusion of nitrogen-containing compounds such as taurine and L-carnitine, which were absent in Brands 1 and 2. Meanwhile, Brand 2 incorporated additional plant-derived additives like corn syrup, corn oil, and soy lecithin, distinguishing it from other brands. All listed ingredients may contribute in various ways to the total nitrogen content of the products [16,24,29,31]. However, statistical analysis indicated that the differences in TN content among the powdered FSMPs for adults were not significant (*p* = 0.082).

Among the *yoghurt-based FSMPs* (Figure 1E), only products formulated *for adults* were included. Brands 2, 4, and 5 were selected for analysis. The electrophoretic profiles of all three products revealed notable similarities in both the number and intensity of fractions, with each presenting five distinct fractions corresponding to molecular weights of ~18, ~23, ~58, ~106, and ~140 kDa. The contents of Brands 2, 4, and 5 showed several similarities, although differences in specific components were also observed. All brands contained dairy protein-based ingredients, vegetable oils (rapeseed and sunflower oils), maltodextrin, flavorings, and emulsifiers (E 471 and soy lecithin). Various B-vitamins were consistently represented across all brands, including thiamine hydrochloride (B1), folic acid (pteroylmonoglutamic acid), and cyanocobalamin (B12). Differences were primarily observed in the types of proteins used, specific additives, and the presence of functional compounds. While Brands 2 and 4 primarily used general dairy proteins, Brand 5 contained whey proteins and fermented milk components. Additionally, all three brands used carrageenan (E407) as a thickening agent. In summary, although yoghurt-based FSMPs differed in terms of basic macro- and micronutrient compositions, these compositional differences were not reflected in their electrophoretic profiles [16,24,29,31]. Statistical analysis indicated that the TN contents of the examined yoghurt-based FSMPs did not differ significantly (*p* = 0.061).

Several FSMP categories presented analytical challenges due to sample heterogeneity and component segregation, including yogurt-based products, powdered formulas, and certain liquid formulations. Conventional nitrogen determination methods, such as the Kjeldahl and Dumas techniques, have been reported to yield ambiguous results in these matrices because of protein aggregation, variable solubility, and the presence of non-protein nitrogen additives. By contrast, the microchip gel electrophoresis approach provided high-resolution separation of protein fractions and reproducible total nitrogen quantification, demonstrating a clear advantage in complex and heterogeneous FSMP matrices. For example, in a yogurt-based FSMP containing both aggregated and soluble proteins, our method delivered consistent and interpretable nitrogen profiles, effectively resolving protein fractions and ensuring reliable quantification in this challenging formulation.

### 2.3. Comparison of FSMPs of the Same Consistency for Newborns and Adults

**Figure 2:**
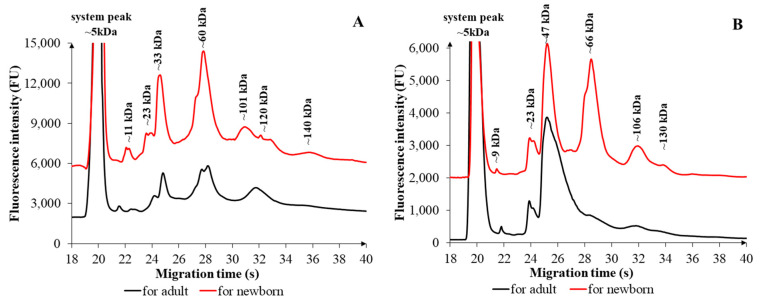
Electrophoretic comparison of FSMPs designed for adults (black line) and newborns (red line) with the same consistency. (**A**) illustrates liquid FSMPs, while (**B**) shows powdered variants. Experimental conditions are described in Section 3.

Figure 2 illustrates the electrophoretic comparison of FSMPs with similar consistencies, demonstrating compositional and additive-related nitrogen differences between formulations intended for newborns and adults.

Figure 2A illustrates the comparison of *liquid FSMP formulations*, demonstrating substantial differences between products formulated for infants and adults. The *liquid FSMP formulated for infants* exhibited a total of seven distinct fractions, with approximate molecular weights of ~11, ~23, ~33, ~60, ~101, ~120, and ~140 kDa. In contrast, the FSMPs for adults displayed six fractions with fewer peaks that only partially overlapped in molecular weight. These differences were observed not only in the number of peaks but also in their fluorescent intensities, likely reflecting variations in additive content employed by the manufacturers. The contents of the products showed several similarities and marked differences, reflecting the distinct nutritional requirements of the two target groups (Supplemental Table S2). Both formulations contain milk-derived components: the infant formula includes lactose, skimmed milk, and a whey-based preparation, whereas the adult formula utilizes milk protein. Plant oils and emulsifiers were also common, with lecithin present in the infant formulation, and soy lecithin and E 471 found in the adult product. Vitamin B12 was consistently present in both formulations. However, differences were observed in carbohydrate and vitamin content. Infant FSMP primarily relied on lactose as a carbohydrate source, whereas the adult product contained maltodextrin. Furthermore, adult FSMP contained B vitamins, including thiamine (B1) and folic acid, which were either absent or present in lower quantities in the infant formulation. Fish oil was a unique component of the infant FSMP, while added flavoring agents were exclusive to the adult formulations. Each of these compositional additives may contribute to the total nitrogen content, either directly (e.g., proteins and amino acids) or indirectly (e.g., nitrogen-containing vitamins and emulsifiers) [16,24,29,31]. A statistically significant difference in TN content was observed between liquid FSMPs for newborns and adults (*p* = 0.041), underscoring the impact of additive composition and protein complexity on the nitrogen profile.

The electropherograms presented in Figure 2B illustrate both the similarities and differences between *powdered FSMPs formulated for newborns and adults*. In the infant formula, six major fractions were identified in the electrophoretic profile, corresponding to the following molecular weights: ~9, ~23, ~47, ~66, ~106, and ~130 kDa. Differences were observed in both the number and intensity of peaks, suggesting distinct compositions and concentrations between the two categories. Both products contained dairy-derived components (milk and milk protein) and functional nitrogen-containing compounds (Supplemental Table S3), such as L-carnitine and B-vitamins, including thiamine hydrochloride (B1), folic acid, and cyanocobalamin (B12). Powdered FSMP intended for infants had a considerably more complex formulation, including milk, demineralized whey, whey concentrate, milk protein, and fish oil. In contrast, the adult formulation was limited to milk and milk proteins as primary protein sources. Additionally, the infant formula contained a variety of vegetable oils (e.g., high-oleic sunflower oil, coconut oil, rapeseed oil, and *Mortierella alpina* oil), none of which were found in the adult FSMP. Other differences included the presence of L-tryptophan and soy lecithin (used as emulsifiers) exclusively in the infant FSMP. Conversely, the adult formulation included taurine, which was absent in infant products. These ingredients may contribute to the total nitrogen content [16,24,29]. Despite these compositional differences, no statistically significant difference was observed in the TN content between powdered FSMPs intended for infants and adults (*p* = 0.911).

The composition of the two formulations developed for different target groups and presented in different physical forms revealed several similarities, alongside numerous significant differences that reflect the distinct age-related and physiological needs of the respective populations. Overall, it can be stated that powdered and liquid FSMPs share common features regarding their basic macronutrient components (e.g., milk protein source, vegetable oil, and emulsifier). However, target group-specific differences were evident in terms of the applied vitamin profiles, carbohydrate sources, and certain functional components.

### 2.4. Statistical Diversity in FSMPs

**Figure 3:**
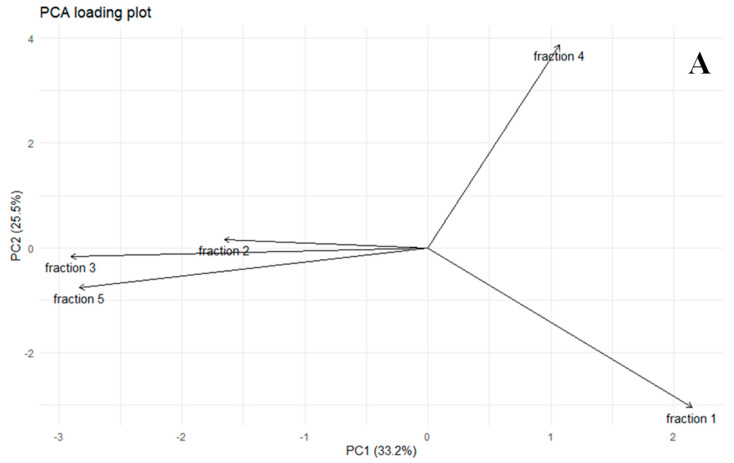

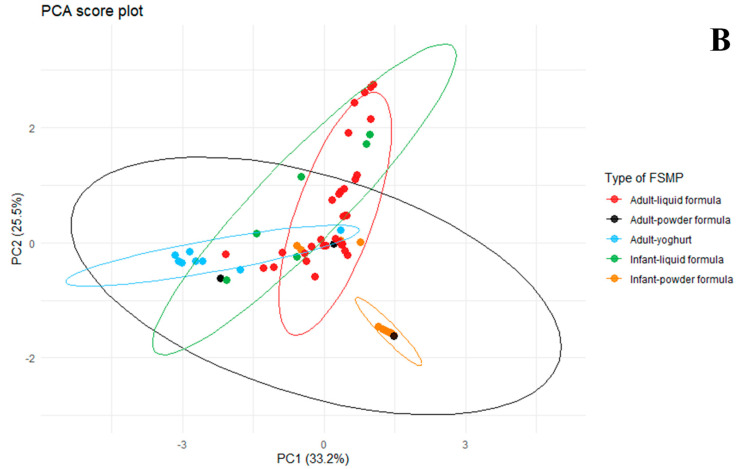
A principal component analysis (PCA) was conducted to evaluate compositional variation among different FSMP types by analyzing the percentage of total time-corrected areas of electrophoretic fractions. (**A**) displays the loading plot, representing the relationships among fractions in terms of their contributions to the first (PC1) and second (PC2) principal components. (**B**) shows the score plot, where individual FSMP samples—classified by type and target population—are projected onto the two-dimensional PCA space. A 95% confidence ellipse is overlaid to highlight group separation and variance.

Figure 3 illustrates the statistical diversity in the percentage of total time-corrected areas of electrophoretic fractions of FSMPs across at least three brands per product category.

The first and second principal components together explained 58.7% of the total variance, with PC1 accounting for 33.2% and PC2 for 25.5%. The loading plot revealed that the first and fourth fractions loaded strongly and positively on PC1, whereas the second fraction contributed significantly to PC2. In contrast, the third and fourth fractions contributed minimally to the variance along either axis, indicating limited discriminatory power (Figure 3A).

The score plot highlights distinct clustering patterns among FSMP formulations. Notably, powdered infant formulas (orange dots) were clearly separated from both adult liquid FSMPs (red dots), infant liquid FSMPs (green dots), and adult yoghurt-based FSMPs (light blue dots). These separations suggest substantial differences in protein content and nitrogen-contributing components across FSMP types and target age groups. Adult powdered FSMPs (black dots) exhibited the highest degree of intra-group variability, reflecting brand-specific differences in the formulation (Figure 3B).

## 3. Materials and Methods

### 3.1. Nutritional Formulas

Nutritional formulas were collected between 2023 and 2024 from Petz Aladár County Teaching Hospital in Győr and the Department of Paediatrics, Medical School, University of Pécs. These products are commercially available in Hungary, packaged in either carton packs or PET plastic bottles, which do not require refrigeration before opening, and typically contain various additives such as sugars, flavorings, emulsifiers, oils, and other supplementary ingredients. The samples collected from the hospitals represent the full range of standard nutritional formula brands that were available in Hungary at the time of the study. Products designed for specific medical conditions (e.g., metabolic disorders, allergies) were not included. Additionally, the study did not aim to compare different flavor variants within the same brand. A total of eighty-eight product samples, representing fifty-five brands, were collected across five FSMP categories: liquid formulas for newborns (4 brands), liquid formulas for adults (29 brands), powdered formulas for newborns (13 brands), powdered formulas for adults (3 brands), and yoghurt-based formulas for adults (6 brands). Most products were available with at least two different expiry dates, allowing for broader sampling within each category. Following collection, the unopened products were randomized, homogenized at room temperature, and aliquoted into 10 mL sealable test tubes. Three aliquots were prepared for each product, and all samples were stored at −20 °C until analysis. Analyses were performed in duplicate for each product.

### 3.2. Microchip Gel Electrophoresis for Electrophoretic Profiling and Total Nitrogen Determination

For rapid and efficient analysis of proteins in nutritional formulas, microchip gel electrophoresis was performed using the Agilent 2100 Bioanalyzer with the High Sensitivity Protein 250 (HSP 250, Agilent Technologies, Santa Clara, CA, USA) method, based on size separation of proteins, as previously described [16,39]. Briefly, liquid and yoghurt-based products were centrifuged at 8500 rpm for 10 min, and the samples were taken from the protein-enriched middle phase. Powdered products were reconstituted in distilled water to achieve a nominal protein concentration of 1 mg/mL. From each sample, 4.5 µL was mixed with 0.5 µL of standard labeling buffer (SLB) to adjust the pH to 8–9, followed by the addition of 0.5 µL of fluorescent dye, according to the manufacturer’s protocol [40]. The samples were then incubated in the dark at room temperature for 10 min. The fluorescent dye contains a proprietary NHS-ester functional group that covalently binds to primary amines of nitrogen-containing compounds, predominantly the ε-amino groups of lysine residues in proteins. The labeling reaction was carried out under alkaline conditions to facilitate efficient covalent coupling and subsequently quenched with ethanolamine to quench excess reactivity and generate a system peak. Each labeled sample was diluted with distilled water twenty and fifty times, based on the nominal protein concentration, to achieve a final concentration below 0.5 g/100 mL. Then, 5.0 µL of the diluted samples was mixed with 2.5 µL of denaturing solution and incubated at 100 °C for 5 min, followed by centrifugation. Denaturation was performed in the presence of SDS, which imparts a uniform negative charge-to-mass ratio to all proteins, enabling size-based separation. Protein separation was performed using microchips preloaded with a PDMA-based gel matrix, optimized for resolution in the 5–250 kDa range. A total of 6 µL of each denatured sample was loaded into designated wells, along with a molecular weight ladder containing standard proteins. Samples were injected at 1000 V for 80 s (~40 pL injection volume), and separation was conducted for 60 s at 30 °C under an electric field of ~230 V/cm. Detection was achieved by laser-induced fluorescence using an excitation wavelength of 630 nm, emission wavelength of 680 nm, and a red laser diode (λ_max_ = 635 nm). Three parallel measurements were performed for each sample. The results were analyzed using Agilent 2100 Expert software v. B.02.09., with manual qualitative and quantitative evaluation. The molecular weights were determined using calibration curves. The relative percentages of TN in each sample were calculated from time-corrected area (TCA) values [37]. The TN content of the samples was derived from the fluorescent signal intensity of ε-amino group binding, as the applied dye selectively binds to nitrogen-containing residues. Since the labeling procedure captures both protein-based and non-protein nitrogen compounds (e.g., amino acids, peptides, stabilizers), the method provides a comprehensive estimation of TN. No separate total protein (TP) assay was performed; instead, the electrophoretic method was used for simultaneous profiling and TN quantification.

### 3.3. Statistical Analysis

The percentage values derived from time-corrected areas were analyzed using the IBM SPSS Statistics software, version 28.0 (IBM Corp., Armonk, NY, USA). Prior to statistical testing, data distribution was assessed for normality, which was subsequently not confirmed. Accordingly, all variables were reported as median values accompanied by interquartile ranges. The Wilcoxon signed-rank test was used to evaluate the paired differences between the total protein content and the corresponding measured TN content. The Kruskal–Wallis non-parametric one-way ANOVA, followed by post hoc multiple pairwise comparisons, was applied to compare TN content both across expiration dates within each FSMP brand (to assess intra-brand variability) and between different brands within the same FSMP category (to examine inter-brand differences). Statistical significance was set at a threshold of *p* < 0.05.

To explore the variability in nitrogen content across FSMP categories, principal component analysis (PCA) with varimax rotation was conducted using the R statistical environment (version 4.3.2; R Core Team, 2023; R Foundation for Statistical Computing, Vienna, Austria).

## 4. Conclusions

The microchip gel electrophoresis HSP 250 method proved to be a reliable and versatile tool for determining total nitrogen content and electrophoretic profiles in FSMP products across various consistencies and target groups. Compared to conventional TN and TP quantification methods (such as Kjeldahl or Dumas), this technique requires smaller sample volumes, offers rapid results, and provides enhanced resolution for detecting protein fractions. Additionally, the method benefits from a lower detection limit due to fluorescent labeling and avoids the use of hazardous chemicals commonly required in traditional nitrogen determination methods. This further enhances its suitability for routine quality control and clinical laboratory settings, offering a safer, more environmentally friendly, and more sensitive alternative. These advantages allow for more precise characterization of nitrogen-containing components, including non-protein additives, which are often overlooked by conventional assays.

Our findings highlight significant compositional variability between FSMP products based on physical form and target population, with clinically relevant discrepancies between declared and measured TN content. From a clinical and nutritional perspective, these results emphasize the importance of accurate nitrogen labeling, particularly for patients with renal insufficiency, for whom protein and nitrogen intake must be carefully managed. Providing detailed nitrogen content on FSMP product labels would support the development of personalized dietary interventions, help minimize metabolic waste accumulation, and contribute to the overall effectiveness and safety of nutritional therapies for vulnerable patient populations.

Although this study focused on products collected in Hungary, future research aims to include FSMPs available across the European Union to improve the representativeness and generalizability of the findings. Despite its advantages, the microchip gel electrophoresis method has some limitations. The fluorescent dye specifically binds to ε-amino groups, meaning that only nitrogen from amino-containing compounds is directly measured, potentially overlooking other nitrogenous substances. Sample preparation is critical to avoid matrix effects and ensure accurate results.

## Figures and Tables

**Table 1:** Comparison of measured total nitrogen (TN) content and nominal total protein (TP) concentrations in various FSMPs of different consistencies and target groups. Data are presented as median values with interquartile ranges (IQR). Each FSMP type includes at least three brands, with a minimum of two products per brand, each measured in duplicate. Statistically significant *p*-values are indicated in bold.

FSMP Type	Measured TN (g/100 mL)	Nominal TP * (g/100 mL)	*p*-Value
Liquid for newborns (*n* = 16)	9.3 (4.1–23.4)	1.9–2.9	**0.011**
Liquid for adults (*n* = 116)	21.2 (9.4–45.2)	4.0–10.0	**<0.001**
Powder for newborns (*n* = 52)	10.9 (7.3–54.8)	1.3–15.3	**0.001**
Powder for adults (*n* = 12)	75.6 (38.8–81.4)	0.0–7.5	**0.026**
Yoghurt for adults (*n* = 24)	3.9 (3.7–12.6)	7.5–9.4	0.506

* **Nominal TP**: Label-declared total protein concentration (min–max range across different brands).

## Data Availability

The original contributions presented in this study are included in the article/Supplementary Material. Further inquiries can be directed to the corresponding author.

## Supplementary Materials

The following supporting information can be downloaded at: https://www.mdpi.com/article/10.3390/ijms26178233/s1.

## References

[1] European Commission (2017). Information from European Union Institutions, Bodies, Offices and Agencies Commission Notice on the Classification of Food for Special Medical Purposes.

[2] European Parliament (2011). Regulation (EU) No 1169/2011 of the European Parliament and of the Council of 25 October 2011 on the Provision of Food Information to Consumers.

[3] Auer F., Jarvas G., Guttman A. (2021). Recent advances in the analysis of human milk oligosaccharides by liquid phase separation methods. J. Chromatogr. B.

[4] Bakshi S., Paswan V.K., Yadav S.P., Bhinchhar B.K., Kharkwal S., Rose H., Kanetkar P., Kumar V., Al-Zamani Z.A.S., Bunkar D.S. (2023). A comprehensive review on infant formula: Nutritional and functional constituents, recent trends in processing and its impact on infants’ gut microbiota. Front. Nutr..

[5] Chatchatee P., Nowak-Wegrzyn A., Lange L., Benjaponpitak S., Chong K.W., Sangsupawanich P., van Ampting M.T.J., Oude Nijhuis M.M., Harthoorn L.F., Langford J.E. (2022). Tolerance development in cow’s milk–allergic infants receiving amino acid–based formula: A randomized controlled trial. J. Allergy Clin. Immunol..

[6] Kouwenhoven S.M.P., Muts J., Finken M.J.J., van Goudoever J.B. (2022). Low-Protein Infant Formula and Obesity Risk. Nutrients.

[7] Tinghäll Nilsson U., Lönnerdal B., Hernell O., Kvistgaard A.S., Jacobsen L.N., Karlsland Åkeson P. (2024). Low-Protein Infant Formula Enriched with Alpha-Lactalbumin during Early Infancy May Reduce Insulin Resistance at 12 Months: A Follow-Up of a Randomized Controlled Trial. Nutrients.

[8] Liotto N., Orsi A., Menis C., Piemontese P., Morlacchi L., Condello C.C., Giannì M.L., Roggero P., Mosca F. (2018). Clinical evaluation of two different protein content formulas fed to full-term healthy infants: A randomized controlled trial. BMC Pediatr..

[9] Wang Y., Zou Y., Wang J., Ma H., Zhang B., Wang S. (2020). The Protective Effects of 2’-Fucosyllactose Against *E. coli* O157 Infection Are Mediated by the Regulation of Gut Microbiota and the Inhibition of Pathogen Adhesion. Nutrients.

[10] Feng P., Fuerer C., McMahon A., Arendse K., Chanady A., Chen H., Chen W., Ding X., Gao T., Huang K. (2018). Quantification of Whey Protein Content in Milk-Based Infant Formula Powders by Sodium Dodecyl Sulfate–Capillary Gel Electrophoresis (SDS-CGE): Multilaboratory Testing Study, Final Action 2016.15. J. AOAC Int..

[11] Tamura Y., Omura T., Toyoshima K., Araki A. (2020). Nutrition Management in Older Adults with Diabetes: A Review on the Importance of Shifting Prevention Strategies from Metabolic Syndrome to Frailty. Nutrients.

[12] Pellegrino L., Hogenboom J.A., Rosi V., Sindaco M., Gerna S., D’Incecco P. (2022). Focus on the Protein Fraction of Sports Nutrition Supplements. Molecules.

[13] Gaytán-González A., Ocampo-Alfaro M.D.J., Torres-Naranjo F., González-Mendoza R.G., Gil-Barreiro M., Arroniz-Rivera M., López-Taylor J.R. (2020). Dietary Protein Intake Patterns and Inadequate Protein Intake in Older Adults from Four Countries. Nutrients.

[14] Li Q., Wang J. (2025). The Application and Mechanism Analysis of Enteral Nutrition in Clinical Management of Chronic Diseases. Nutrients.

[15] Baik S.M., Kim M., Lee J.G. (2024). Comparison of Early Enteral Nutrition Versus Early Parenteral Nutrition in Critically Ill Patients: A Systematic Review and Meta-Analysis. Nutrients.

[16] Balogh-Hartmann F., Páger C., Bufa A., Sipos Z., Dávidovics A., Verzár Z., Marosvölgyi T., Makszin L. (2024). Comprehensive Study of Total Nitrogen Content and Microfluidic Profiles in Additive-Enriched Plant-Based Drinks. Foods.

[17] Blazek V., Caldwell R.A. (2009). Comparison of SDS gel capillary electrophoresis with microfluidic lab-on-a-chip technology to quantify relative amounts of 7S and 11S proteins from 20 soybean cultivars. Int. J. Food Sci. Technol..

[18] Bleakley S., Hayes M. (2017). Algal Proteins: Extraction, Application, and Challenges Concerning Production. Foods.

[19] Cavallo G., Lorini C., Garamella G., Bonaccorsi G. (2021). Seaweeds as a “Palatable” Challenge between Innovation and Sustainability: A Systematic Review of Food Safety. Sustainability.

[20] Gravel A., Dubois-Laurin F., Turgeon S.L., Doyen A. (2024). The role of the 7S/11S globulin ratio in the gelling properties of mixed β-lactoglobulin/pea proteins systems. Food Hydrocoll..

[21] Hernández H., Nunes M.C., Prista C., Raymundo A. (2022). Innovative and Healthier Dairy Products through the Addition of Microalgae: A Review. Foods.

[22] Nawaz M.A., Singh T.K., Stockmann R., Jegasothy H., Buckow R. (2021). Quality Attributes of Ultra-High Temperature-Treated Model Beverages Prepared with Faba Bean Protein Concentrates. Foods.

[23] Patil U., Benjakul S. (2018). Coconut Milk and Coconut Oil: Their Manufacture Associated with Protein Functionality. J. Food Sci..

[24] Roland I.S., Aguilera-Toro M., Nielsen S.D.-H., Poulsen N.A., Larsen L.B. (2023). Processing-Induced Markers in Proteins of Commercial Plant-Based Drinks in Relation to Compositional Aspects. Foods.

[25] Kiani A.K., Dhuli K., Donato K., Aquilanti B., Velluti V., Matera G., Iaconelli A., Connelly S.T., Bellinato F., Gisondi P. (2022). Main nutritional deficiencies. J. Prev. Med. Hyg..

[26] Castanys-Muñoz E., Martin M.J., Prieto P.A. (2013). 2’-fucosyllactose: An abundant, genetically determined soluble glycan present in human milk. Nutr. Rev..

[27] Turck D., Bohn T., Castenmiller J., De Henauw S., Hirsch-Ernst K.I., Maciuk A., Mangelsdorf I., McArdle H.J., Naska A., Pelaez C. (2022). Safety of 2’-fucosyllactose (2’-FL) produced by a derivative strain (APC199) of Corynebacterium glutamicum ATCC 13032 as a novel food pursuant to Regulation (EU) 2015/2283. EFSA J..

[28] Yao Q., Fan L., Zheng N., Blecker C., Delcenserie V., Li H., Wang J. (2022). 2’-Fucosyllactose Ameliorates Inflammatory Bowel Disease by Modulating Gut Microbiota and Promoting MUC2 Expression. Front. Nutr..

[29] Borsatto J.V.B., Maciel E.V.S., Cifuentes A., Lanças F.M. (2023). Online Extraction Followed by LC–MS/MS Analysis of Lipids in Natural Samples: A Proof-of-Concept Profiling Lecithin in Seeds. Foods.

[30] Quezada C., Urra M., Mella C., Zúñiga R.N., Troncoso E. (2024). Plant-Based Oil-in-Water Food Emulsions: Exploring the Influence of Different Formulations on Their Physicochemical Properties. Foods.

[31] Maltodextrin. https://www.acs.org/molecule-of-the-week/archive/m/maltodextrin.html.

[32] Cecchi S., Di Stante S., Belcastro S., Bertuzzi V., Cardillo A., Diotallevi L., Grabocka X., Kulurianu H., Martello M., Nastasi V. (2023). Supplemented Very Low Protein Diet (sVLPD) in Patients with Advanced Chronic Renal Failure: Clinical and Economic Benefits. Nutrients.

[33] Gonzalez P., Lozano P., Solano F. (2022). Unraveling the Metabolic Hallmarks for the Optimization of Protein Intake in Pre-Dialysis Chronic Kidney Disease Patients. Nutrients.

[34] Garneata L., Mocanu C.-A., Simionescu T.P., Mocanu A.E., Dragomir D.R., Mircescu G. (2024). Low Protein Diet Reduces Proteinuria and Decline in Glomerular Filtration Rate in Advanced, Heavy Proteinuric Diabetic Kidney Disease. Nutrients.

[35] Sofoulaki E., Tzanakakis V.A., Giannopoulos G., Kapellakis I., Kabourakis E., Chatzistathis T., Monokrousos N. (2023). Different Contribution of Olive Groves and Citrus Orchards to Soil Organic Carbon Sequestration: A Field Study in Four Sites in Crete, Greece. Sustainability.

[36] Mæhre H., Dalheim L., Edvinsen G., Elvevoll E., Jensen I.-J. (2018). Protein Determination—Method Matters. Foods.

[37] Agilent Technologies, Inc (2016). Quantification Strategies Using the High Sensitivity Protein 250 Assay for the Agilent 2100 Bioanalyzer Technical Note 5989-8941EN.

[38] Agilent Technologies, Inc (2019). Protein Analysis with the Agilent 2100 Bioanalyzer System An Overview of the Protein Assay Portfolio.

[39] Balogh-Hartmann F., Páger C., Bufa A., Madarászné Horváth I., Verzár Z., Marosvölgyi T., Makszin L. (2023). Microfluidic Analysis for the Determination of Protein Content in Different Types of Plant-Based Drinks. Molecules.

[40] Agilent Technologies, Inc (2008). Agilent High Sensitivity Protein 250 Kit Guide.

