# Supplementary material for: A Microfluidic Approach for Profiling Total Nitrogen Content in Age-Specific Nutritional Formulas Using Microchip Gel Electrophoresis
Source: Int J Mol Sci. 2025 Aug 25;26(17):8233. doi: 10.3390/ijms26178233 (PMC12427775; doi:10.3390/ijms26178233)
Supplement: Supplementary file 1 [file ijms-26-08233-s001.zip › ijms-3786643-supplementary.pdf]

A Microfluidic Approach for Profiling Total Nitrogen Content in Age-Specific Nutritional Formulas Using Microchip Gel Electrophoresis

Fruzsina BALOGH-HARTMANN [1], Csilla PÁGER [1], Anna DÁVIDOVICS [2], Sára NAGY[3], Tamás MAROSVÖLGYI [1] and Lilla MAKSZIN [1,*]

[1] Institute of Bioanalysis, Medical School, Szentágothai Research Center, University of Pécs, 7624 Pécs, Hungary; fruzsina.hartmann@aok.pte.hu (F.B.-H.); csilla.pager@aok.pte.hu (C. P.); marosvolgyi.tamas@pte.hu (T.M.); lilla.makszin@aok.pte.hu (L.M.)

[2] Department of Languages for Biomedical Purposes and Communication, University of Pécs, Medical School, 7624 Pécs, Hungary, anna.davidovics@aok.pte.hu (A.D.)

[3] Doctoral School of Health Sciences, Faculty of Health Sciences, University of Pécs, Pécs, Hungary, nagy.sara@edu.pte.hu (S.N.)

*Corresponding author: lilla.makszin@aok.pte.hu

Supplementary Table S1. Protein and non-protein nitrogen (NPN) additives identified in FSMP brands analyzed in this study, grouped by five product types (liquid FSMPs for newborns, liquid FSMPs for adults, powder FSMPs for newborns, powder FSMPs for adults, yoghurt FSMPs for adults). These additives are classified into seven categories contributing to total nitrogen (TN) content.

LIQUID FOR NEWBORNS	MILK-BASED PROTEINS	PLANT-BASED COMPONENTS	AMINO ACIDS/ AMINO ACIDS DERIVATIVES	OILS/FATS	VITAMINS	OLIGOSACCHARIDES, DISACCHARIDES / NUCLEOTIDES	FOAMING AGENT/ SWEETENERS/ EMULSIFIERS
**Brand 2**	whey protein (from cow's milk)	vegetable oils (corn oil), oil from *Mortierella alpina*	taurine, L-carnitine	fish oil, anhydrous milk fat, medium-chain triglycerides (from coconut and palm oil)	thiamine hydrochloride (vitamin B_1), pteroylmonoglutamic acid (folic acid / vitamin B_9), cyanocobalamin (vitamin B_{12})	lactose (from cow's milk), cytidine 5′-monophosphate, adenosine 5′-monophosphate, uridine 5′-monophosphate sodium salt, inosine 5′-monophosphate sodium salt, guanosine 5′-monophosphate sodium salt	maltodextrin
**Brand 3**	whey protein hydrolysate	vegetable oils (corn oil, rapeseed oil, sunflower oil), oil from *Mortierella alpina*	L-histidine, taurine, L-tyrosine, L-arginine, L-carnitine	medium-chain triglycerides (from coconut and/or palm oil) fish oil	thiamine hydrochloride (vitamin B_1), pteroylmonoglutamic acid (folic acid / vitamin B_9), cyanocobalamin (vitamin B_{12})	cytidine 5′-monophosphate, adenosine 5′-monophosphate, uridine 5′-monophosphate sodium salt, inosine 5′-monophosphate sodium salt, guanosine 5′-monophosphate sodium salt	maltodextrin
**Brand 4**	16% skimmed milk whey preparation (from milk)	vegetable oils (soybean, sunflower, coconut, and rapeseed oil)	-	fish oil	vitamin B12 (cobalamin)	lactose (from cow's milk)	lecithin

Liquid for Adults	milk-based proteins	Plant-based components	amino acids/ amino acids derivatives	oils/fats	Vitamins	Oligosaccharides, disaccharides / Nucleotides	Foaming agent/ sweeteners/ Emulsifiers
**Brand 21**	whey protein (from cow's milk)	vegetable oils	-	-	thiamine hydrochloride (vitamin B_1), pteroylmonoglutamic acid (folic acid / vitamin B_9), cyanocobalamin (vitamin B_{12})	-	maltodextrin, aroma, soy lecithin
**Brand 23**	whey protein (from cow's milk)	vegetable oils	-	fish oil	thiamine hydrochloride (vitamin B_1), pteroylmonoglutamic acid (folic acid / vitamin B_9), cyanocobalamin (vitamin B_{12})	cytidine 5′-monophosphate, adenosine 5′-monophosphate, uridine 5′-monophosphate sodium salt, inosine 5′-monophosphate sodium salt, guanosine 5′-monophosphate sodium salt	maltodextrin, aroma, soy lecithin, sweeteners
**Brand 26**	whey protein (from cow's milk)	vegetable oils (sunflower, rapeseed oil)	-	fish oil	thiamine hydrochloride (vitamin B_1) pteroylmonoglutamic acid (folic acid / vitamin B_9)	lactose (from cow's milk)	maltodextrin, aroma (strawberry), soy lecithin, acesulfame-K

Powder for newborns	milk-based proteins	Plant-based components	amino acids/ amino acids derivatives	oils/fats	Vitamins	Oligosaccharides, disaccharides / Nucleotides	Foaming agent/ sweeteners/ Emulsifiers
**Brand 1**	whey protein hydrolysate	vegetable oils (Coconut oil, rapeseed oil, Sunflower oil), oil from *Mortierella alpina*	L-tyrosine, L-carnitine, taurine	fish oil	thiamine hydrochloride (vitamin B_1), pteroylmonoglutamic acid (folic acid / vitamin B_9),	cytidine 5′-monophosphate, adenosine 5′-monophosphate, uridine 5′-monophosphate sodium salt, inosine 5′-monophosphate sodium salt, guanosine 5′-monophosphate sodium salt	soy lecithin
**Brand 3**	skimmed milk, whey product (from milk), whey protein (from milk)	vegetable oils (coconut oil, rapeseed oil, sunflower oil), cereal flakes (from rice)	L-tryptophan L-carnitine	fish oil	thiamine hydrochloride (vitamin B_1), pteroylmonoglutamic acid (folic acid / vitamin B_9), cyanocobalamin (vitamin B_{12})	cytidine 5′-monophosphate, adenosine 5′-monophosphate, uridine 5′-monophosphate sodium salt, inosine 5′-monophosphate sodium salt, guanosine 5′-monophosphate sodium salt	soy lecithin
**Brand 5**	casein, skimmed milk, demineralized whey (from milk), whey protein concentrate, whey protein	vegetable oils (palm, coconut, rapeseed, sunflower oil, high-oleic sunflower oil)	L-tryptophan, L-carnitine, taurine	fish oil	thiamine hydrochloride (vitamin B_1), pteroylmonoglutamic acid (folic acid / vitamin B_9), cyanocobalamin (vitamin B_{12})	lactose (from cow's milk), cytidine 5′-monophosphate, adenosine 5′-monophosphate, uridine 5′-monophosphate sodium salt, inosine 5′-monophosphate sodium salt, guanosine 5′-monophosphate sodium salt	soy lecithin, carob bean gum

Powder for adults	milk-based proteins	Plant-based components	amino acids/ amino acids derivatives	oils/fats	Vitamins	Oligosaccharides, disaccharides / Nucleotides	Foaming agent/ sweeteners/ Emulsifiers
**Brand 1**	-	-	-	-	-	-	maltodextrin
**Brand 2**	casein content	corn oil	-	-	thiamine hydrochloride (vitamin B_1), pteroylmonoglutamic acid (folic acid / vitamin B_9), cyanocobalamin (vitamin B_{12})	-	corn syrup, soy lecithin
**Brand 3**	milk, milk protein	-	L-carnitine, taurine	-	thiamine hydrochloride (vitamin B_1), pteroylmonoglutamic acid (folic acid / vitamin B_9), cyanocobalamin (vitamin B_{12})	-	-

Yoghurt for adults	milk-based proteins	Plant-based components	amino acids/ amino acids derivatives	oils/fats	Vitamins	Oligosaccharides, disaccharides / Nucleotides	Foaming agent/ sweeteners/ Emulsifiers
**Brand 2**	milk protein	vegetable oils (rapeseed oil, sunflower oil)	-	-	thiamine hydrochloride (vitamin B_1), pteroylmonoglutamic acid (folic acid / vitamin B_9), cyanocobalamin (vitamin B_{12})	-	maltodextrin, flavorings, soy lecithin, carrageenan
**Brand 4**	milk protein	vegetable oils (rapeseed oil, sunflower oil)	-	-	thiamine hydrochloride (vitamin B_1), pteroylmonoglutamic acid (folic acid / vitamin B_9) cyanocobalamin (vitamin B_{12})	-	maltodextrin, carrageenan, flavorings, soy lecithin
**Brand 5**	whey protein, fermented skimmed milk powder	vegetable oils	-	-	thiamine hydrochloride (vitamin B_1), pteroylmonoglutamic acid (folic acid / vitamin B_9), cyanocobalamin (vitamin B_{12})	-	maltodextrin, flavorings, soy lecithin, carrageenan, lactic acid from milk

Supplemental Table S2 The content of adult and infant liquid FSMPs, represented in Figure 2A, classified into seven categories, reveals both similarities and notable differences. Differences are highlighted in bold.

	MILK-BASED PROTEINS	PLANT-BASED COMPONENTS	AMINO ACIDS/ AMINO ACIDS DERIVATIVES	OILS/FATS	VITAMINS	OLIGOSACCHARIDES, DISACCHARIDES / NUCLEOTIDES	FOAMING AGENT/ SWEETENERS/ EMULSIFIERS
LIQUID FOR NEWBORNS	***skimmed milk whey preparation (from milk)***	vegetable oils (soybean, sunflower, coconut, and rapeseed oils, in varying proportions) (20%)	-	***fish oil***	vitamin B_{12}	***lactose (from milk)***	lecithin
LIQUID FOR ADULTS	***milk protein***	vegetable oils	-	-	vitamin B_{12}	-	soy lecithin, ***E471, flavor***

Supplemental Table S3 The content of adult and infant powder FSMPs, represented in Figure 2B, classified into seven categories, reveals both similarities and notable differences. Differences are highlighted in bold.

	MILK-BASED PROTEINS	PLANT-BASED COMPONENTS	AMINO ACIDS/ AMINO ACIDS DERIVATIVES	OILS/FATS	VITAMINS	OLIGOSACCHARIDES, DISACCHARIDES / NUCLEOTIDES	FOAMING AGENT/ SWEETENERS/ EMULSIFIERS
POWDER FOR NEWBORNS	***skimmed milk, demineralized whey, whey concentrate,*** milk protein	***plant oils (high oleic sunflower oil, coconut oil, rapeseed oil, sunflower oil)***	***L-tryptophan,*** L-carnitine	**fish oil, *Mortierella alpina* oil**	vitamin B_{12}	-	***soy lecithin***
POWDER FOR ADULTS	***milk,*** milk protein	-	***taurine,*** L-carnitine	-	***folic acid,*** vitamin B_{12}	-	-
